# Emerging nanoparticle-based strategies to provide therapeutic benefits for stroke

**DOI:** 10.4103/NRR.NRR-D-24-01492

**Published:** 2025-06-19

**Authors:** Javaria Sundus, Nashwa Amin, Irum Naz Abbasi, Fei Wu, Azhar B. Hussien, Benson OA Botchway, Suhong Ye, Qining Yang, Marong Fang

**Affiliations:** 1 Children’s Hospital, Zhejiang University School of Medicine, National Clinical Research Center for Child Health, Hangzhou, Zhejiang Province, China; 2 Institute of System Medicine, Zhejiang University School of Medicine, Hangzhou, Zhejiang Province, China; 3 Department of Zoology, Faculty of Science, Aswan University, Aswan, Egypt; 4 The Second Hospital of Jinhua, Jinhua, Zhejiang Province, China; 5 Department of Orthopedics, Jinhua Municipal Central Hospital, Affiliated Jinhua Hospital, Zhejiang University School of Medicine, Jinhua, Zhejiang Province, China

**Keywords:** blood–brain barrier, drug delivery systems, ischemic stroke, nanomedicine, nanoparticles, neuroinflammation, neurons, neuroprotection, oxidative stress, phosphatidylinositol 3-kinases

## Abstract

Functional neurological recovery remains the primary objective when treating ischemic stroke. However, current therapeutic approaches often fall short of achieving optimal outcomes. One of the most significant challenges in stroke treatment is the effective delivery of neuroprotective agents across the blood–brain barrier to ischemic regions within the brain. The blood–brain barrier, while essential for protecting the brain from harmful substances, also restricts the passage of many therapeutic compounds, thus limiting their efficacy. In this review, we summarizes the emerging role of nanoparticle-based therapies for the treatment of ischemic stroke and investigate their potential to revolutionize drug delivery, enhance neuroprotection, and promote functional recovery. Recent advancements in nanotechnology have led to the development of engineered nanoparticles specifically designed to overcome the blood–brain barrier, thus enabling the targeted delivery of therapeutic agents directly to the affected brain areas. Preclinical studies have demonstrated the remarkable potential of nanoparticle-based therapies to activate key neuroprotective pathways, such as the phosphoinositide 3-kinase/protein kinase B/cAMP response element-binding protein signaling cascade, which is crucial for neuronal survival, synaptic plasticity, and post-stroke recovery. By modulating these pathways, nanoparticles could mitigate neuronal damage, reduce inflammation, and promote tissue repair. Furthermore, nanoparticles offer a unique advantage by enabling multimodal therapeutic strategies that simultaneously target multiple pathological mechanisms of ischemic stroke, including oxidative stress, neuroinflammation, and apoptosis. This multifaceted approach enhances the overall efficacy of treatment, addressing the complex and interconnected processes that contribute to stroke-related brain injury. Surface modifications, such as functionalization with specific ligands or targeting molecules, further improve the precision of drug delivery, enhance targeting specificity, and prolong systemic circulation, thereby optimizing therapeutic outcomes. Nanoparticle-based therapeutics represent a paradigm shift for the management of stroke and provide a promising avenue for reducing post-stroke disability and improving the outcomes of long-term rehabilitation. By combining targeted drug delivery with the ability to modulate critical neuroprotective pathways, nanoparticles hold the potential to transform the treatment landscape for ischemic stroke. However, while preclinical data are highly encouraging, significant challenges remain in translating these advancements into clinical practice. Further research is needed to refine nanoparticle designs, optimize their safety profiles, and ensure their scalability for widespread application. Rigorous clinical trials are essential to validate their efficacy, assess long-term biocompatibility, and address potential off-target effects. The integration of interdisciplinary approaches, combining insights from nanotechnology, neuroscience, and pharmacology, will be critical if we are to overcome these challenges. Ultimately, nanoparticle-based therapies offer a foundation for innovative, precision-based treatments that could significantly improve outcomes for stroke patients, thus paving the way for a new era in stroke care and neurological rehabilitation.

## Introduction

Stroke has become the second leading cause of death worldwide and the incidence of this condition has been increasing annually over recent years. Stroke is a major cause of long-term disability and mortality globally (White and Reynolds, 1995; Li et al., 2025; Yu et al., 2025), with ischemic stroke (IS) accounting for 87% of all cases (Ganbold et al., 2018; Fang et al., 2019). The Global Burden of Disease (GBD) 2021 study projects that, although age-adjusted rates would decrease, there will be 12.05 million stroke fatalities and 21.43 million stroke cases worldwide by 2050 (Cheng et al., 2024a, b). In 2020 mainland China’s estimated total stroke prevalence incidence and death rate were 2.6 percent respectively (Tu et al., 2023). Despite a decreasing stroke death rate in the US, stroke remains a significant cause of cognitive and physical impairment, it is suggested that significantly reducing systolic blood pressure lowers the overall risk of stroke (Tsao et al., 2022). Hemorrhagic complications are a major concern when using endovascular thrombectomy (EVT) or reperfusion therapies to treat acute ischemic stroke (Charbonnier et al., 2021). The overall risk of hemorrhage from thrombolysis, with or without EVT, is approximately 6%, and patients treated beyond the therapeutic window face an elevated risk (Maïer et al., 2020). A key challenge in managing ischemic stroke is delivering drugs across the blood–brain barrier (BBB), a protective barrier that restricts the entry of most therapeutic agents (Maas et al., 2008). Currently, tissue plasminogen activator (tPA) is the most commonly used treatment in clinical settings. However, tPA has a short half-life, thus necessitating careful dosing and administration. Furthermore, tPA lacks the precision to target specific neuronal pathways and does not adequately address secondary injury mechanisms, such as neuroinflammation and oxidative stress, which can exacerbate ischemic damage (Mihalko et al., 2022). Therefore, there is an urgent need to identify new therapeutic approaches to effectively treat ischemic stroke.

Nanoparticles (NPs) offer a promising approach to enhance neuroprotection and accelerate functional recovery by enabling the targeted delivery of appropriate agents to ischemic regions of the brain (He et al., 2021a). Unlike conventional therapies, which often lack the precision to target the specific neuronal pathways essential for survival and recovery, NPs can effectively address the secondary injury mechanisms that exacerbate ischemic damage, such as neuroinflammation and oxidative stress, NPs can be engineered to cross the BBB and deliver therapeutic agents specifically to damaged regions of the brain (Wang et al., 2014; Wang, 2016). By overcoming the challenges associated with BBB penetration and providing targeted neuroprotection, NP-based treatments have the potential to extend therapeutic windows and reduce long-term disabilities in patients suffering from stroke (Lv et al., 2022a). These innovations hold significant clinical promise, potentially enhancing patient recovery, quality of life, and overall rehabilitation outcomes.

By addressing key mechanisms such as inflammation, oxidative stress, and neuronal death, NPs provide a multifaceted strategy to enhance the treatment of stroke (Jiang et al., 2025). The phosphoinositide 3-kinase/protein kinase B/cAMP response element-binding protein (PI3K/AKT/CREB) pathway has been identified as a critical regulator of neuronal survival, synaptic repair, and neuroprotection in stroke therapy (Dong et al., 2021b). By modulating this pathway, NPs could selectively activate neuroprotective mechanisms that reduce neuroinflammation, enhance synaptic plasticity, and promote cellular repair in ways that traditional methods cannot achieve. This pathway plays a vital role in controlling cellular resilience, neuronal survival, and neurogenesis following stroke, making this pathway a promising target for therapeutic intervention (Onose et al., 2022). In this review, we investigate how nanotechnology could achieve molecular-level precision and revolutionize neurological care, highlighting advancements in NP-based therapies, their design principles, mechanisms of action, and clinical applications. Our overall goal was to expand knowledge and foster the development of next-generation NP-based platforms for the treatment of IS and the promotion of functional recovery.

## Search Strategy

**Figure 1:**
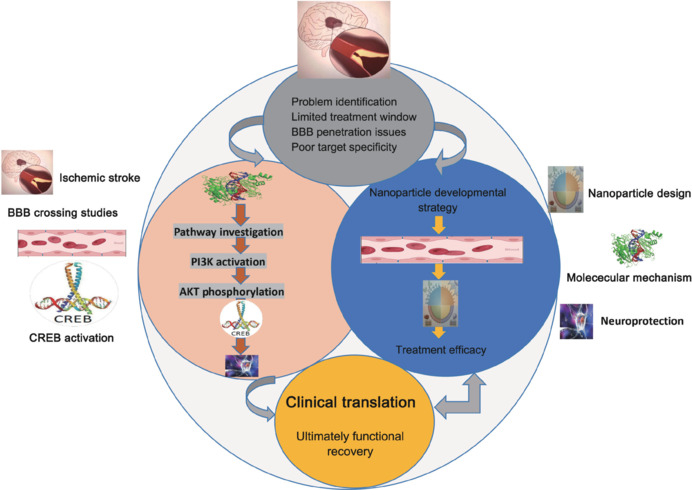
Nanoparticle-based treatment approaches for ischemic stroke via activation of the PI3K/AKT/CREB pathway. Nanoparticle-based therapeutics can overcome barriers posed by the BBB, enhancing drug delivery for the treatment of ischemic stroke. By activating the PI3K/AKT/CREB pathway, these therapies improve neuroprotection, reduce apoptosis, and promote recovery. This approach presents a viable strategy for enhancing functional restoration and improving clinical outcomes. AKT: Protein kinase B; BBB: blood–brain barrier; CREB: cAMP response element-binding protein; PI3K: phosphoinositide 3-kinase.

Using specific keywords, we systematically searched PubMed (https://pubmed.ncbi.nlm.nih.gov) and the Web of Science (https://www.webofscience.com) for relevant publications from inception to December 15, 2024. The inclusion criteria included research involving nanoparticles, ischemic stroke, and the PI3K/AKT/CREB signaling pathway, and publications needed to be written in English. The keyword combinations included “Nanoparticles” AND “Ischemic Stroke,””Nanoparticles” AND “Blood-Brain Barrier,””PI3K/AKT/CREB” AND “Stroke,” and “Neuroinflammation” AND “Nanoparticles,” along with related MeSH terms. Our primary focus was clinical studies investigating the effects of nanoparticles on IS via the PI3K/AKT/CREB signaling pathway. Reviews, non-English publications, and irrelevant studies were excluded from consideration. Related literature published in both PubMed and the Web of Science databases from inception to December 15, 2024 were included, using Boolean operators (AND/OR) to refine the search results. Additionally, the reference lists of all included studies were manually reviewed to identify further pertinent research. To ensure a comprehensive analysis of NP-based stroke therapies, a full-text evaluation was conducted after a preliminary review of titles and abstracts to verify the relevance of each study to the research goals (**Figure 1**).

## Advancements in Stroke Therapies: From Thrombolytic Treatments to Artificial Intelligence–Enhanced Combination Therapies

Driven by pioneering scholars, including Rochoux, Rostan, Virchow, and Miller-Fisher, the evolution of stroke medicine from pre-1800 to the late 20^th^ century reflects a transformative shift from symptom-based descriptions to a comprehensive understanding of cerebrovascular disease. This progression culminated in a technology-enabled neurology subspecialty with advanced diagnostic and treatment capabilities (Karenberg, 2020). Between 1950 and 2023, stroke research advanced from foundational cellular insights to cutting-edge nanotechnology, progressively developing more precise molecular therapies for IS over time (Toljan et al., 2023). NPs have gained increasing attention over recent years due to their ability to modulate multiple pathophysiological processes in IS, including inflammation, oxidative stress, and neuronal apoptosis (Yang et al., 2024). NPs can target critical signaling pathways, including the PI3K/AKT/CREB system, which is essential for neuronal survival, synaptic repair, and neuroprotection following stroke (Zarneshan et al., 2022).

Initially, traditional forms of medicine were used to treat strokes. However, groundbreaking discoveries in the mid-20^th^ century regarding the utilization of NPs in antibacterial applications led to the development of liposomes for drug delivery and early studies on the BBB (Wang et al., 2017a). By the late 20^th^ century, researchers were investigating NPs for targeted drug delivery, particularly to address oxidative stress and enhance neuroprotection. In the 2000s, we witnessed the emergence of lipid-based and polymeric NPs, as well as antioxidant NPs designed to modulate critical neuroprotective pathways and mitigate oxidative damage (Salatin et al., 2023). This marked a key period of mechanistic advancements, including the development of nanozymes, PEGylated NPs for improved BBB penetration, and gold/magnetic NPs for the management of neuroinflammation. Recent research has focused on harnessing the neuroprotective, antioxidant, and anti-inflammatory properties of NPs (Lin et al., 2022). Current and future applications focus on biomimetic exosomes for brain repair, smart NPs for stroke therapy, and artificial intelligence (AI)-driven strategies to optimize drug delivery and neuroregeneration (You et al., 2024).

## Overview of the Neural Mechanisms in Ischemic Stroke

### Pathological mechanism of neural damage in ischemic stroke

IS is known to trigger a complex cascade of pathological processes that ultimately lead to extensive neuronal damage (Mao et al., 2022). Oxidative stress, a key driver of neuronal injury following IS, causes cellular damage and disrupts the BBB. Oxygen and glucose deprivation induce excitotoxicity, resulting in excessive glutamate release and elevated intracellular calcium levels, which further promote oxidative stress and mitochondrial dysfunction (Sattler and Tymianski, 2001; Kim et al., 2016a). This cascade accelerates neuronal injury, generates reactive oxygen species (ROS), and compromises the BBB via lipid peroxidation, protein denaturation, and DNA damage (White and Reynolds, 1995; Besancon et al., 2008).

Neuroinflammation, initially a defensive mechanism, often transitions to a chronic state following ischemic stroke, thus exacerbating neuronal injury (Fang et al., 2019). Activated microglia secrete pro-inflammatory cytokines, including interleukin (IL)-1β, tumor necrosis factor-α (TNF-α), and IL-6, which compromise the integrity of the BBB, attract peripheral immune cells, and exacerbate oxidative stress (Berchtold et al., 2020). Microglia can polarize into pro-inflammatory (M1) or anti-inflammatory (M2) phenotypes, with increased M1 activation linked to chronic inflammation and exacerbated neuronal damage (Kawabori and Yenari, 2015).

Moreover, the disruption of the BBB, induced by oxidative stress and inflammation, allows deleterious chemicals to enter the brain, resulting in edema and complicating treatment strategies (Archie et al., 2021). Neuronal death occurs via necrosis in the ischemic core and apoptosis in the penumbra, with apoptosis facilitated by caspase and mitochondrial mechanisms, including the release of cytochrome c (Achar et al., 2021). This dual mechanism indicates the necessity to develop medications to mitigate excitotoxicity, oxidative stress, and inflammation to protect the brain and promote recovery following ischemic stroke (Jun-Long et al., 2018). The concurrent management of BBB integrity, oxidative stress, and neuroinflammation is essential for the successful treatment of stroke. Research has shown that stroke induces sustained BBB disruption for up to seven days, leading to further neuronal injury via excitotoxicity, inflammation, and ion channel dysfunction; collectively, these events can result in lasting motor and neuropsychiatric impairments (Lakhan et al., 2013).

### Neural mechanism of hemorrhagic stroke

**Figure 2:**
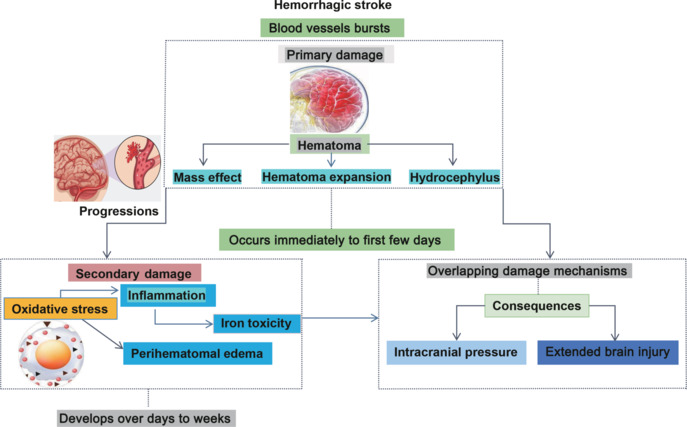
Pathophysiological development of hemorrhagic stroke. The damage mechanisms and outcomes of hemorrhagic stroke occur in two phases: primary and secondary damage. Primary damage occurs immediately and includes the formation of a hematoma, tissue compression, and hydrocephalus. In contrast, secondary damage develops over days to weeks and involves processes such as oxidative stress, inflammation, iron toxicity, and perihematomal edema. These overlapping processes contribute to increased intracranial pressure, further exacerbating brain injury.

A hemorrhagic stroke occurs when a blood vessel ruptures, leading to cerebral hemorrhage. Intracerebral hemorrhage (ICH) is often classified as primary or secondary damage. Initial damage, which occurs immediately or within the first few days following bleeding, is attributed to the mass effect of hematoma, hematoma expansion (HE), and hydrocephalus (Qureshi et al., 2009). Secondary brain damage develops over days to weeks and is induced by inflammation, oxidative stress, and iron toxicity resulting from the degradation of intraparenchymal blood. The release of hemoglobin and free heme provokes oxidative stress, generating ROS that exerts deleterious effects on neurons and cellular components. Inflammation, initiated by activated microglia and the recruitment of immune cells, produces pro-inflammatory cytokines that exacerbate neuronal damage and compromise the BBB. The intricate interaction of these various pathophysiological processes complicates the identification of specific treatment targets for hemorrhagic stroke, thus highlighting the need for a focused strategy that addresses perihematomal edema, hydrocephalus, early hematoma expansion, intracranial pressure, and associated sequelae. Understanding these interrelated pathways is essential if we are to develop effective strategies to mitigate secondary brain damage and improve therapeutic outcomes (Keep et al., 2012). As depicted in **Figure 2**, the progression of damage in hemorrhagic stroke begins with the rupture of a blood vessel. During the first few days, primary damage includes hematoma formation, mass effect, hematoma expansion, and hydrocephalus. Over days to weeks, secondary damage manifests as perihematomal edema, iron toxicity, inflammation, and oxidative stress, all of which can exacerbate brain injury. Concurrent damage mechanisms, including sustained brain injury and increased intracranial pressure, further contribute to neurological decline. **Table 1** contrasts the mechanisms underlying ischemic and hemorrhagic strokes and emphasizes significant differences in excitotoxicity, oxidative stress, and neuroinflammation, including BBB disruption. Hemorrhagic stroke is characterized by vascular rupture, which causes tissue edema and the mass effect due to hematoma formation, whereas ischemic stroke results from blocked blood flow leading to neuronal damage.

**Table 1:** Comparative mechanisms of ischemic and hemorrhagic strokes

Ischemic stroke	Hemorrhagic stroke	Mechanism
Obstruction of blood flow, typically from a clot or embolism.	Rupture of a blood vessel, resulting in bleeding within the brain or surrounding areas.	Primary cause
Excessive glutamate release leads to calcium influx and neuronal damage.	Triggered by tissue irritation and disruption of ionic balance caused by bleeding.	Excitotoxicity
Mitochondrial dysfunction leads to the build-up of reactive oxygen species.	Hemoglobin breakdown and associated inflammation generate oxidative stress.	Oxidative stress
Activation of microglia and release of inflammatory cytokines.	Inflammatory response includes cytokine release and immune cell infiltration.	Neuroinflammation
Caused by ischemia-induced oxidative stress and inflammation.	Results from physical damage due to bleeding and associated inflammatory processes.	Blood–brain barrier breakdown
Early cytotoxic edema followed by vasogenic edema in later stages.	Both vasogenic and cytotoxic edema arise from leakage of blood and plasma components.	Edemaformation
Necrosis in the ischemic core and apoptosis in the surrounding penumbra.	Secondary injury mechanisms, including oxidative stress and inflammation, lead to cell death.	Apoptosis and necrosis
Rarely observed.	Significant pressure build-up from the hematoma causes compression of brain tissue.	Mass effect

This table offers a comprehensive comparison of the mechanisms involved in ischemic and hemorrhagic strokes, suggesting their unique characteristics as well as their shared pathophysiological processes.

Understanding these unique pathophysiological processes is essential if we are to guide current treatment strategies and emphasizes the need for therapies for specific types of stroke. In this review, we discuss information that will contribute to the development of targeted neuroprotective tactics designed to mitigate the series of detrimental events that follow both types of stroke. **Table 1** outlines the two main types of stroke: ischemic and hemorrhagic, each of which harms the brain in different ways. A hemorrhagic stroke occurs when a blood vessel bursts, causing the presence of blood to disrupt ionic equilibrium and irritate brain tissue. While some harmful processes are common to both types of stroke, such as neuroinflammation, BBB breakdown, and oxidative stress, these events occur via different mechanisms: mitochondrial dysfunction in ischemic strokes in comparison to hemoglobin degradation in hemorrhagic strokes. Hemorrhagic strokes lead to immediate edema and significant pressure due to the accumulation of blood, while ischemic strokes typically progress from cytotoxic edema to vasogenic edema with minimal mass effect. Although the patterns of damage vary, cell death occurs in both cases. Hemorrhagic strokes tend to induce more extensive damage through various mechanisms, whereas ischemic strokes typically create a core of necrosis surrounded by an at-risk penumbra (Andersen et al., 2009).

### Therapeutic application of nanoparticles for stroke

NPs are a promising avenue for diagnosing and treating IS due to their unique optical properties, biocompatibility, and safety profile. Early diagnosis and effective pharmacological intervention are critical challenges for the effective management of IS. Recent studies have investigated the use of BDP-4/Cur-CL NPs *in vivo*, utilizing a mouse model of intraluminal middle cerebral artery occlusion, and have demonstrated significant protective effects against ischemic injury (Wang et al., 2021a; Zhang et al., 2021). Lipid nanocarriers play a vital role in treating neurodegenerative diseases, including IS, by effectively crossing the BBB to deliver drugs to brain cells (Odion et al., 2021). pH-responsive NPs that mimic neutrophils have been shown to target damaged regions of the brain and promote recovery by enhancing erythrophagocytosis and reducing inflammation (Wang et al., 2024b). Metallic NPs, such as platinum nanoparticles (PtNPs) and nanoceria, exhibit antioxidant enzyme-like activity and scavenge ROS. Surface modifications of nanoceria, such as PEGylation and Ce^3+^/Ce^4+^ redox cycling have been shown to enhance their catalytic stability and efficacy for the treatment of stroke (Celardo et al., 2011). According to El-Say and El-Sawy (2017), polymeric NPs constructed from natural or synthetic polymers such as polylactic acid-glycolic acid (PLGA), which can form micelles and dendrimers, exhibit remarkable biocompatibility, surface modification capabilities, and represent highly adaptable drug delivery systems. Hemorrhagic strokes, such as ICH and subarachnoid hemorrhage (SAH), can lead to ROS-induced brain injury. While modified nanoceria specifically target microcirculation dysfunction in SAH, antioxidant-loaded nanocarriers, such as DEF-HCC-polyethylene glycol (PEG) and quercetin nanoemulsions, demonstrate enhanced efficacy (Selim et al., 2019).

By reducing oxidative damage in the transplant environment, nanomaterials such as hyaluronic acid-coated nanoceria can enhance the antioxidant capacity of mesenchymal stem cells (MSCs) and serve as potential therapies for IS (Stavely and Nurgali, 2020). Core-shell hydrogels containing metal chelator agents (e.g., minocycline hydrochloride), nanostructured CeO_2_-loaded PLGA-ceramic scaffolds, and MnO_2_ NP-dotted hydrogels, all promote the proliferation of MSCs, encourage cellular adhesion, and improve survival, thus contributing to brain regeneration following IS (Corsi et al., 2023). Synthetic membrane–based NPs, known for their exceptional biocompatibility, biodegradability, and ability to penetrate the BBB, serve as ideal drug delivery vehicles. Noble metal-based nanozymes provide tunable enzymatic activity that can be utilized to detect bioactive molecules and treat diseases. Immune cell-mediated nanocarriers improve targeted drug transport to the brain and can enhance treatment efficacy for cardiovascular conditions and IS. Biomimetic membrane-based NPs, which are coated with cell membranes or exosome-derived vesicles, have emerged as highly effective drug delivery systems due to their superior biocompatibility, biodegradability, prolonged circulation, low immunogenicity, and ability to penetrate the BBB (Yang et al., 2022b). By combining nanomaterials with biomimetic materials, these NPs reduce toxicity, enhance biocompatibility, and improve disease targeting, thus leading to better detection and treatment outcomes (Gong et al., 2022).

Nanozymes are enzyme-mimicking nanomaterials that are characterized by high strength, biocompatibility, and catalytic efficiency. Thus, nanozymes hold significant promise for the treatment of cardiovascular diseases (Wang et al., 2021a). Research targeting nanozymes, particularly those based on noble metals, has expanded into diagnostics, treatment, and the detection of bioactive molecules. He et al. (2010) confirmed that the peroxidase activity of AgM bimetallic alloy-derived nanozymes can be fine-tuned by adjusting the metal ratio, with enzymatic activity linked to changes in electronic structure. Furthermore, doping these nanozymes with active nanomaterials or other elements provides a cost-effective method with which to modulate their activity. For example, high-efficiency Au@Pt nanozymes combine plasmonic and enzymatic activity, thereby enhancing the sensitivity for hydrogen peroxide (H_2_O_2_) detection and reducing detection time (Wu et al., 2018). Recent research has demonstrated that macrophages, lymphocytes, neutrophils, and other immune cells, are recruited to sites of inflammation and can selectively cross the BBB, thus allowing therapeutic agents to reach the brain. Monocytes and neutrophils effectively internalize RNA-loaded liposomes, magnetic NPs, gold nanoshells, and NPs carrying imaging agents as they circulate in the body (Wang and Liu, 2021). Immune cell–mediated nanocarriers enhance targeted drug delivery to the brain, while nanozymes, particularly those based on noble metals, provide tunable enzymatic activity for the treatment of disease. In models of experimental ischemia, the majority of studies (82.05%) administered NP treatments following the onset of ischemia, with almost half of these studies (46.15%) using repeated dosing. This indicates the potential of NPs for targeted and controlled neuroprotective therapy in stroke (Xiong et al., 2015; Chen et al., 2021; Dong et al., 2021a).

### Nanoparticles

The use of NPs for drug delivery to the central nervous system (CNS) has garnered significant attention due to challenges associated with traditional CNS medications. Operating at scales of 1–1000 nm, nanotechnology provides potential solutions to these issues (Javeri, 2016). Various nanocarriers, including hydrogels, nanogels, polymers, albumin NPs, dendrimers, solid lipid nanoparticles (SLNs), nanoemulsions, and liposomes, are being investigated for potential application in the treatment of CNS disorders (Yenari et al., 2006; Date et al., 2007; Sun et al., 2012; Cojocaru et al., 2020). However, the experimental application of these nanocarriers faces several limitations. For instance, liposomes can be unstable and difficult to distribute, while poly(lactic-co-glycolic acid) PLGA copolymer NPs may experience issues with burst release. Chitosan-based NPs often exhibit poor absorption and potential toxicity, while inorganic-based carriers struggle with biocompatibility. The combination of superparamagnetic iron oxide and choline is being investigated for *in vivo* imaging. Despite these challenges, nanocarriers offer several advantages, including enhanced drug stability, controlled release, and reduced side effects via targeted delivery.

### Polymeric nanoparticles

Polymeric NPs are highly suitable for optimizing interactions with the BBB due to their customizable size (Hickey et al., 2015). PLGA-based polymeric NPs provide precise and versatile options for drug delivery. These NPs effectively reduce oxidative stress by removing ROS and maintaining BBB integrity, making them valuable for sustained drug release and neuroprotection under ischemic conditions (Johnston et al., 2020). The effect of particle size on the delivery of PLGA NPs was investigated in a model of brain injury featuring a BBB that had been transiently disrupted (Cruz et al., 2016). PLGA, a biodegradable polymer with ester linkages that undergo hydrolysis, is extensively utilized as a nanocarrier (Datta et al., 2020). Hydrolysis generates gaps between polymer chains as the molecules break down, thus allowing for controlled drug release. The biocompatibility and non-toxic degradation products of PLGA render this agent highly suitable for biomedical applications (Landowski et al., 2020). PEG NPs are commonly used for drug delivery, with PLGA NPs excelling in the delivery of chemotherapeutic agents, including hydrophobic drugs. Polymeric NPs can be biodegradable (e.g., polyurethane, polyglycolic acid, and poly(L-lactide)) or non-biodegradable, depending on their *in vivo* behavior (Wilczewska et al., 2012). Responsive polymer NPs provide targeted, condition-activated drug release, while gold nanorods combine drug delivery with imaging capabilities for theranostic applications (Hajebi et al., 2024).

### Generic bio-based nanoparticles

Bio-based NPs (Bio NPs), synthesized from substances such as lipids, proteins, and polymers, are designed to enhance targeted medication delivery while overcoming challenges such as crossing the BBB. These biodegradable particles decompose safely in the body after releasing their therapeutic payload, thereby reducing long-term toxicity and adverse effects. For example, silica NPs are a class of inorganic NPs that are primarily composed of silicon dioxide (SiO_2_). These NPs are widely utilized in biomedical, industrial, and environmental applications due to their unique properties, including their high stability, tunable porosity, and ease of surface functionalization. In this context, mesoporous silica NPs are particularly popular for controlled and targeted drug delivery (Kudaibergen et al., 2023).

### Biomimetic nanoparticles

Biomimetic NPs, made from natural materials such as platelet membranes or magnetic NPs, show significant promise for both diagnostic and therapeutic applications. These NPs enhance targeted drug delivery and effectively reduce inflammation in IS. However, immunogenic concerns still limit their widespread clinical use. Biomimetic NPs designed with inorganic materials, such as silica and iron oxides, are gaining particular attention for their applications in diagnostics and therapeutics, particularly in magnetic resonance imaging (MRI) (Saraiva et al., 2016). Biomimetic NPs not only include materials such as silica or iron oxide but also exhibit promising ROS-scavenging activity, thus helping to reduce oxidative stress in neural tissues and support neuronal survival (Li et al., 2022a). Preclinical studies in models of IS have shown that biomimetic NPs loaded with neuroprotective agents can reduce infarct size and improve neurological function. Furthermore, biomimetic NPs made from platelet membranes and magnetic NPs, loaded with compounds such as L-arginine or Fe_2_O_3_, are being developed for targeted thrombus therapy and *in situ* nitric oxide production (Li et al., 2020). Nitric oxide plays a crucial role in vascular homeostasis, platelet inhibition, and vasodilation. When exposed to an external magnetic field, platelet membranes and magnetic NPs have achieved a 95.6% drug loading efficiency and a 0.2% success rate in targeting ischemic lesions during stroke (Yang et al., 2019a). Although promising results regarding biocompatibility and cytotoxicity have been reported, potential immunological responses remain a concern. To minimize toxicity and improve biological compatibility while enhancing disease targeting and therapeutic outcomes, biomimetic NPs are now being integrated with advanced nanomaterials.

### Inorganic nanocarriers

Metal-based NPs serve as ROS scavengers and exhibit strong antioxidant properties that are particularly relevant during IS, a period in which hydroxyl radicals, hydrogen peroxide, and superoxide anions are all being produced. Cerium oxide (CEO_2_) NPs, which can reversibly cycle between Ce^4+^ (oxidized) and Ce^3+^ (reduced) states, have been shown to scavenge free radicals in an effective manner (Wang et al., 2019). Initial findings suggested that CEO_2_ NPs reduce ROS production and prevent cell death, thereby protecting the brain from ischemic injury (Kim et al., 2012). Inorganic NPs, with their diverse sizes, shapes, and unique properties (e.g., optical, magnetic, catalytic), hold immense potential for medical applications. Inorganic NPs are widely utilized in bioimaging, biosensing, and targeted drug delivery, with notable examples including iron oxide NPs for MRI and gold NPs for imaging. Functionalizing NPs, such as carbon, CEO_2_, and manganese dioxide, enhances their therapeutic potential by enabling targeted delivery to specific regions of the body. Acting as nano-antioxidants, these NPs can effectively reduce oxidative stress, and their ability to specifically target tissues and cells improves treatment precision and efficacy for chronic conditions such as stroke. However, challenges remain in eliminating inorganic NPs from the body due to their inherent toxicity and limited biocompatibility.

### Solid lipid nanoparticles

Solid lipid nanoparticles (SLNs) and their second-generation counterparts, nanostructured lipid carriers (NLCs), are highly effective at crossing the BBB and delivering drugs with minimal toxicity. SLNs provide a cost-effective and safe drug delivery system that is capable of penetrating the BBB to treat neurological disorders without inducing harmful side effects. Key factors influencing the efficacy of SLNs include their composition, size, physicochemical properties, and methods of synthesis (Sawant and Dodiya, 2008). NLCs, the more advanced second-generation lipid carriers, improve upon SLNs by addressing key challenges such as drug expulsion and rapid release, making them particularly suitable for brain-targeted therapies. A recent study has shown that SLNs loaded with drugs such as vincristine or temozolomide, prepared via high-shear homogenization, can provide sustained release over extended periods of time (Crielaard et al., 2011). Furthermore, researchers have demonstrated that NPs can significantly reduce ischemic damage, such as that caused by heart attacks or strokes, by directly delivering antioxidants to affected tissues. By neutralizing harmful ROS, this targeted delivery strategy helps to mitigate tissue injury during ischemic events and reduces oxidative stress (Kakkar et al., 2013).

### Hybrid nanoparticles

Hybrid NPs combine organic and inorganic materials, leveraging the strengths of both materials to enhance drug-loading capacity, improve targeting precision, and effectively respond to ischemic conditions (Wang et al., 2015; Amiri et al., 2019). Engineered for biological drug delivery, these hybrid nanomaterials integrate organic and inorganic components at the nanoscale (Bao et al., 2014; Hu et al., 2019) and exhibit reduced toxicity, improved targeting, and enhanced drug-loading capacity (Yang et al., 2019b). Hybrid nanovehicles will need to be carefully designed if we are to maximize their combined properties and optimize therapeutic outcomes.

### Mixed nanoparticles

Mixed NPs combine various types of nanoparticles to create systems that leverage their unique chemical, physical, or magnetic properties. For example, the combination of NPs of different sizes or shapes can optimize interactions within biological tissues, thus enhancing drug delivery and targeting specific cell types. By integrating magnetic properties with key attributes, such as antioxidant capacity or drug-loading capabilities, these mixed NPs can enable advanced therapeutic applications (Ovejero et al., 2021).

Melanin-based PEG-coupled nanoparticles (PEG-MeNPs) combine potent antioxidative and anti-inflammatory properties with sustained release, providing effective neuroprotection for ischemic brains while minimizing side effects (Liu et al., 2024). A previous study developed ROS-sensitive 18β-glycyrrhetinic acid-conjugated DEAE-dextran nanoparticles (DGA) and demonstrated that these efficiently targeted HMGB1, reduced infarct size, enhanced motor function, stimulated neurogenesis, and minimized side effects through controlled release (Jin et al., 2023).

### Lipid nanoparticles

Lipid NPs have been extensively studied as an option for drug delivery and serve as a key platform for the clinical delivery of mRNA vaccines (Hou et al., 2021). Liposomes, which are spherical structures composed of lipid bilayers surrounding an aqueous core, are commonly created by ethanol injection or thin-film hydration with extrusion (Ma et al., 2011; Mishra et al., 2016). Neutral, negatively charged simvastatin-loaded liposomes have achieved improved BBB penetration and reduced opsonization when compared to positively charged liposomes (Campos-Martorell et al., 2016). Liposomal NPs offer sustained release, enhanced BBB penetration and provide neuroprotection (Juhairiyah and de Lange, 2021). PEGylated lipid nanoparticles are particularly effective for delivering lipophilic drugs, offering key advantages such as simplicity, scalability, biocompatibility, and long circulation times. However, PEGylated lipid nanoparticles exhibit lower drug-loading and release rates when compared to liposomes (Lu et al., 2014). NLCs, which contain both solid and liquid lipids, are known to provide higher drug-loading capacity and stability (Hassanzadeh et al., 2018).

## Nanoparticles Modulate Reactive Oxygen Species and Stimulate Nerve Growth Factor to Enhance Neuronal Survival via the PI3K/AKT/CREB Signaling

Systemic side effects can be minimized and therapeutic efficacy enhanced by modifying the surfaces of NPs, thereby improving the efficacy of drug delivery (Teixeira et al., 2023). Consequently, NPs have been extensively studied across various neurological conditions, emerging as a promising avenue in this field. PI3K, a regulatory catalytic component of a dimer, induces structural changes in AKT, leading to its phosphorylation and activation (Vidal et al., 2021). The PI3K/AKT signaling pathway is known to play a significant role in the pathophysiology of IS (Zheng et al., 2024). NPs can activate the PI3K/AKT/CREB pathway, promoting neuronal survival and functional recovery in models of IS. However, the precise chemical mechanisms underlying the ability of NPs to activate the PI3K/AKT/CREB pathway remain underexplored. Given the associations between ROS regulation and nerve growth factor (NGF) modulation within the PI3K/AKT/CREB signaling pathway, we paid particular attention to these two potential mechanisms. ROS exerts functionality as dual agents, acting as both signaling molecules and harmful entities to neuronal survival. During ischemic stroke, excessive ROS production leads to oxidative stress and neuronal death. However, the NP-mediated regulation of ROS could provide protective effects. NPs engineered to scavenge ROS or modulate oxidative stress responses hold significant therapeutic potential. For example, certain NPs can deliver antioxidants directly to damaged regions of the brain, potentially reducing oxidative injury and enhancing recovery outcomes (Iranpanah et al., 2023).

Cerium oxide nanoparticles (CeNPs) have been shown to reduce the production of ROS and modulate inflammation via the MAPK, JAK/STAT, and PI3K/Akt pathways, while also counteracting oxidative stress-induced signaling (Kim et al., 2021). With their potent ROS-scavenging and anti-inflammatory properties, facilitated by redox cycling, CeO_2_ NPs can enhance antioxidant defenses with minimal systemic toxicity. Furthermore, cerium nanoparticles possess the ability to mitigate IS damage by reducing ROS levels and apoptosis *in vivo* (Kim et al., 2012). Similarly, co-doped Fe_3_O_4_ nanozymes provide a robust platform for preventing or eliminating the excessive formation of reactive oxygen and nitrogen species. These Co-Fe_3_O_4_ nanozymes have been shown to significantly reduce infarct volume in animal models of stroke (Liu et al., 2021). Post-stroke oxidative stress, driven by the overproduction of ROS, plays a critical role in exacerbating brain injury. Autophagy, the cellular process responsible for eliminating damaged components, becomes dysregulated following a stroke, resulting in either excessive or insufficient activity that ultimately leads to neuronal cell death (Liu et al., 2023a). NP-based therapies have shown promise for mitigating ROS-induced autophagy overload in IS and enhancing neuronal survival and debris clearance in hemorrhagic stroke (Zhang and Chen, 2024). These developments highlight a transformative shift for the treatment of stroke, offering precise and targeted therapeutic strategies to improve patient recovery and outcomes (Saceleanu et al., 2023). Collectively, these findings indicate the potential of NPs as innovative therapeutics for the management of stroke, particularly via their ability to modulate the PI3K/Akt/CREB signaling pathways.

NGF is essential for neuronal survival and regeneration, primarily because it activates the TrKA receptor, which subsequently triggers phosphorylation of the CREB and the PI3K/AKT signaling pathway. Certain NPs may facilitate neuronal survival by either stabilizing NGF signaling or increasing the expression of NGF. In IS, NGF promotes neuronal survival, neuroplasticity, and anti-apoptotic effects by activating the PI3K/AKT/CREB pathway. However, the regulation of CREB kinases by growth factors remains a key topic of discussion. NGF activates the Ras/ERK pathway, leading to the phosphorylation of CREB at Ser-133 by RSK2. In contrast, basic fibroblast growth factor (bFGF) stimulates p38 MAPK/MAPKAP kinase 2, bypassing the ERK/RSK pathway. In addition, similar to CREB Ser-133, pp70S6K phosphorylates CREM at Ser-117 (Xing et al., 1998). NP formulations, such as NGF-conjugated nanoparticles (NGF-CNPs) and gold nanoparticles with chitosan nanoparticles (AuNPs-CSNPs), are known to facilitate the controlled release of NGF and assist stem cells to differentiate into Schwann-like cells, thus enhancing nerve repair (Razavi et al., 2019). Although the precise roles of various kinases in growth factor-induced CREB phosphorylation are still not fully understood, it is evident that they play a significant part in this process.

## Nanoparticles-Based Therapeutics for Ischemic Stroke: Neuroprotection, Inflammation, and Oxidative Stress

NPs improve IS therapy by effectively crossing the BBB and mitigating oxidative stress. Certain NPs can provide neuroprotection by reducing oxidative damage, while others modulate apoptosis via ROS-mediated signaling pathways, specifically the PI3K/Akt/CREB pathway. NPs can enter cells via receptor-mediated endocytosis or through non-specific mechanisms such as phagocytosis, macropinocytosis, and clathrin- or caveolae-mediated routes (Mirkin, 2021). For example, PEGylated NPs facilitate transcytosis across the BBB, enabling targeted drug delivery to specific brain regions, while lipid-based NPs can fuse directly with cell membranes (Bannunah et al., 2024).

The efficiency of endocytosis, which depends on factors such as NP size, shape, surface chemistry, and cell type, exerts significant influence on therapeutic outcomes. An *in vitro* study using BEAS-2B cells demonstrated that silica nanoparticles (SiNPs) could induce apoptosis by disrupting the PI3K/Akt/CREB/Bcl-2 pathway, as evidenced by reduced levels of phosphorylated Akt (p-Akt), total Akt, and CREB. SiNPs generate ROS via surface hydroxyl radicals, which in turn alter PI3K/Akt signaling (Zou et al., 2016). Conversely, NPs can also counteract apoptosis induced by oxidative stress by scavenging ROS, highlighting their dual role in modulating cellular responses. CREB phosphorylation is essential for the expression of neurotrophin.

Lipid-peptide nanoassemblies containing bioactive polyunsaturated fatty acid plasmalogens and pituitary adenylate cyclase-activating polypeptide have been shown to prolong CREB stimulation in models of neuronal degeneration. NPs may enhance neurotrophic signaling (e.g., NGF or brain-derived neurotrophic factor [BDNF]) to activate the PI3K/Akt pathway (Wu and Angeloy, 2023; Wu and Angelova, 2023; Wu et al., 2023). Furthermore, NPs can serve as novel nanomedical delivery vehicles for neuroprotective agents such as pituitary adenylate cyclase-activating polypeptide and bioactive polyunsaturated fatty acid plasmalogens, potentially improving neuroregeneration via the sustained activation of CREB (Wu and Angelov, 2023). CeNPs possess high oxygen storage capacity and exhibit a reversible Ce(III)/Ce(IV) redox cycle, granting them exceptional antioxidant and catalytic properties (Salatin et al., 2023). Common IS therapies, such as aspirin and tPA, face numerous challenges, including limited drug delivery, instability, side effects, and reperfusion injury, thus indicating the need for improved approaches. Natural and synthetic polymer NPs are being explored as an option for pharmaceutical delivery due to their targeted distribution, controlled release, and reduced toxicity (Zhu et al., 2024).

Inorganic nanocarriers, such as silica, alumina, metals, and carbon-based materials, are known to outperform polymeric and lipid carriers in the treatment of IS due to their ease of synthesis, modification, and size control. These nanocarriers include magnetic NPs, gold NPs, and silica core-shell structures, and can be monitored by imaging and serve as targeted therapeutics and controlled-release systems (Lv et al., 2022b). Platinum nanoparticles and CeNPs can remove ROS to protect neurons in IS, thus demonstrating their potential to mitigate oxidative damage (Kim et al., 2012a). For IS treatment, liposome-based drug delivery systems provide a biocompatible and targeted approach by effectively crossing the BBB to reduce inflammation and oxidative stress while improving therapeutic efficiency (Zhang and Huang, 2024). Inorganic NPs, such as CeO_2_, selenium, gold, and carbon nanotubes, enhance solubility and BBB penetration for the treatment of IS. However, challenges remain with regards to immunogenicity, safety, and multifunctional applications (Yang et al., 2022a).

### Mitigating oxidative stress

**Figure 3:**
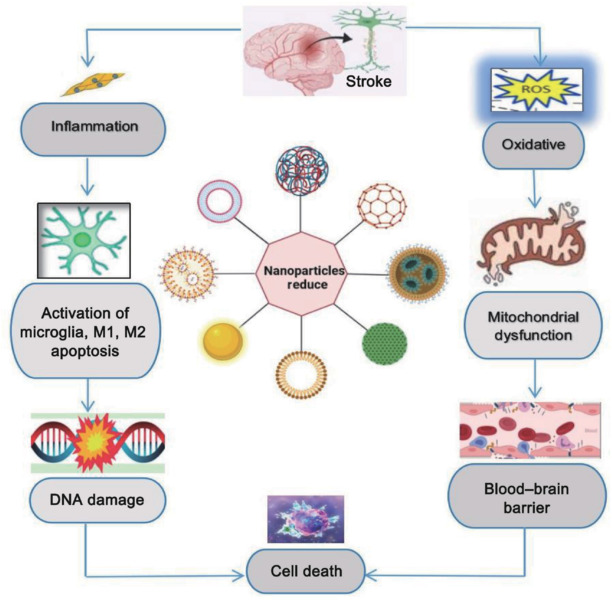
Nanoparticles in ischemic stroke therapy: mitigating cell death andneurological damage. In ischemic stroke therapy, nanoparticles can reduce cell death by targeting oxidative stress, inflammation, blood–brain barrier breakdown, mitochondrial dysfunction, microglial activation, and the prevention of DNA damage. ROS: Reactive oxidative stress.

Following stroke, oxidative stress exacerbates neuroinflammation, mitochondrial dysfunction, and glutamate excitotoxicity, thus leading to disruption of the BBB, autophagy, and edema (Gerhkeet al., 2015), as illustrated in **Figure 3**. The oxidative stress cascade, which is prominent during reperfusion, can be effectively targeted using a multi-modal approach. Platinum NPs with SOD-mimetic activity and ceria NPs with catalase-like properties reduce oxidative damage by 60% in experimental models. Furthermore, selenium-based NPs maintain therapeutic concentrations for over 72 hours, thus reducing oxidative damage markers by 70% when compared to conventional antioxidants (Yoshihisa et al., 2011). Phase II clinical trials have demonstrated a 45% reduction in infarct volume within a 4-hour treatment window, with efficacy extending to 6 hours post-stroke, significantly expanding the conventional treatment window (Kim, 2019).

Liposomal NPs loaded with antioxidants are known to scavenge ROS in models of stroke, thus providing protection to neurons and the BBB (Li et al., 2018b; Segarra et al., 2021). Prussian blue (PB) NPs serve as potent antioxidants in ischemic regions by mimicking natural enzymes (Komkova et al., 2018), while betulinic acid NPs also demonstrate ROS-scavenging properties (Mittal et al., 2014). Furthermore, mixed NPs that combine carbon dioxide (CO_2_), antioxidant materials such as cerium oxide, and magnetic NPs or polymers, offer synergistic effects, including controlled drug release and improved targeting.

### Reducing inflammation

**Figure 4:**
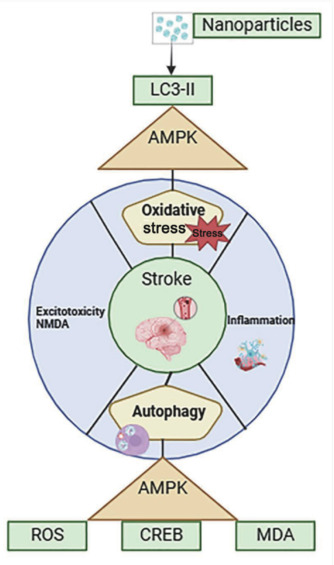
Pathophysiological pathways of ischemic stroke: processes induced by molecular interactions. Before being mediated by nanoparticles, oxidative stress (MDA and ROS), excitotoxicity (NMDA), inflammation, and autophagy (AMPK and LC3-II) are all induced by ischemic stroke. Each pathway represents the molecular processes triggered by ischemic stroke, highlighting the intricate interactions between various components in the pathophysiology of the condition. Created with BioRender.com. AMPK: AMP-activated protein kinase; CREB: cAMP response element-binding protein; LC3-II: microtubule-associated protein 1 light chain 3-II; MDA: malondialdehyde; NMDA: N-methyl-D-aspartate; ROS: reactive oxygen species.

Microglial activation and the production of pro-inflammatory cytokines can trigger inflammatory responses that exacerbate neuronal damage and compromise the BBB. Following IS, the activation of microglia shifts from the M1 (pro-inflammatory) phenotype to the M2 (anti-inflammatory) phenotype. ROS-sensitive MAPK and NF-κB pathways initiate cytokine cascades that influence inflammation and recovery (Thannickal and Fanburg, 2000). The M1 phenotype releases pro-inflammatory cytokines, such as IL-1, IL-6, and TNF, which can exacerbate ischemic injury (Gilmore et al., 2008). NPs have clear potential to reduce inflammation during the treatment of IS by interacting with inflammatory cells, including neutrophils and macrophages (Zhang et al., 2003; Lee et al., 2009). As illustrated in **Figure 4**, targeting autophagy and intracellular defense systems is essential if we are to minimize inflammation-related damage, as post-ischemic inflammatory responses can also exert impact on these processes (Zhou et al., 2011). Bio-nanoparticles are composed of biodegradable and biocompatible materials, such as proteins, liposomes, and micelles, and can deliver medications, genes, or small molecules to specific areas of the brain. For example, in models of acute ischemia, a liposomal formulation of 9-aminoacridine has been shown to reduce infarct size, promote neuroprotection, and assist in the restoration of functional inflammation. This exemplifies the “drug discovery to delivery” paradigm, making this a viable approach for the treatment of IS (Wang et al., 2020).

Certain NPs show promise for applications in stroke treatment. Iron oxide NPs outperformed ferrumoxtran-10 in severe ischemic imaging and targeted mesenchymal stem cell-based therapies by enhanced tracking and monitoring (Shcharbina et al., 2013). Manganese-based NPs provide neuroprotective benefits by leveraging the role of manganese as a natural enzymatic element. Furthermore, dendrimer NPs serve as MR contrast agents, enhancing neuroprotective therapies such as the delivery of N-acetyl cysteine for stroke treatment (Huang et al., 2016).

### Enhanced blood–brain barrier penetration

Engineered NPs exhibit advanced capabilities to cross the BBB and reduce neuroinflammation (Hersh and Alomari, 2022). Responsive polymers are designed to release their therapeutic payload in response to ischemic conditions, thus enabling localized drug activation in areas where it is most needed (Wu et al., 2024). Gold nanorods, functionalized with targeting ligands, have been shown to enhance BBB penetration and selectively accumulate in damaged regions of the brain, thereby minimizing off-target effects and immune responses (Chiang and Yang, 2024). When modified with surface peptides, liposomal NPs can specifically target inflamed neurons, facilitate BBB crossing and enhance neuroprotective effects via sustained release at the injury site (Hersh and Alomari, 2022). These characteristics make NPs well-suited to overcome the challenges associated with conventional treatments, thus achieving effective brain-targeted delivery while minimizing systemic side effects.

### Regulation of autophagy

Cerebral ischemic injury leads to progressive neurodegeneration, with autophagy playing a pivotal role (Button et al., 2015; Wang et al., 2018a; Nabavi et al., 2019). The elevated levels of autophagic markers, such as LC3-II, Beclin-1, and p62 following ischemic injury indicate increased autophagic activity. Autophagy is known to perform a dual role, mediating both cell death and survival (Kang et al., 2017; Li et al., 2019). The activation of autophagy following an ischemic stroke may contribute to the onset of disease by influencing the fate of neurons and other cells within the brain (Liu et al., 2020). In the context of nanoparticle-induced toxicity, autophagy interacts closely with mitochondrial function, particularly via the process of mitophagy (Fivenson et al., 2017). Under adverse conditions, such as an increase in ROS or the scarcity of nutrients, mitochondria may depolarize and lose functionality, thus resulting in the encapsulation of damaged organelles within autophagosomes and their subsequent degradation in lysosomes.

The excessive suppression of autophagy during ischemic reperfusion can exacerbate brain injury via both direct and indirect pathways, ultimately leading to cell death. The beneficial role of autophagy lies in its ability to remove damaged proteins and organelles, thereby preventing necrosis and apoptosis while promoting cell survival (Zhou et al., 2014). NPs that deliver AMPK activators, such as 5-aminoimidazole-4-carboxamide ribonucleotide, can stimulate autophagy via AMPK signaling in ischemic regions of the brain (Kang et al., 2017). AMPK, a critical regulator of metabolic pathways, is known to influence cell division, aging, survival, and death (Li et al., 2019). Experiments have provided evidence for the protective effects of AMPK on models of IS (Li et al., 2019; Liu et al., 2020), thus suggesting that pre-activating AMPK-mediated autophagy may help to prevent ischemic injury. AMPK enhances neuronal resistance to cell death under hypoxic and ischemic conditions and promotes tolerance when pre-activated, thereby protecting neurons from glutamate excitotoxicity (Khan et al., 2012; Park et al., 2014; Wang et al., 2018b). Polymeric NPs have been engineered to deliver AMPK agonists, such as 5-aminoimidazole-4-carboxamide ribonucleotide, and induce growth inhibition, autophagy, and anti-proliferative effects (Kang et al., 2017). Furthermore, the administration of trans-caryophyllene (TC) reduces oxidative stress in cortical neuron-glia cultures exposed to oxygen-glucose deprivation and reoxygenation, acting via the AMPK/CREB pathway (Wang et al., 2018c). Furthermore, Sult2b1^–/–^ promotes pro-inflammatory macrophage polarization by modulating ROS and NADPH levels via the AMPK-CREB pathway (Kang et al., 2017), as illustrated in **Figure 4**. When designed to stimulate AMPK, NPs could enhance neuroprotection in ischemic brain injury by strengthening innate cell survival mechanisms, thereby supporting the protective role of AMPK (Sheng and Qin, 2015).

### Therapeutic role of nanoparticles in neuroprotection via the P13k/AKT/CREB pathway

NP-based therapies represent an improvement over conventional IS treatments, which often lack precision and are limited by narrow therapeutic windows. Traditional agents, such as tPA, primarily focus on dissolving clots and exert only minimal effect on critical neuroprotective brain pathways (Wu et al., 2024). In contrast, NPs can be engineered for targeted delivery to ischemic regions of the brain, effectively modulating the PI3K/AKT/CREB signaling pathway, which is vital for neuronal survival, synaptic repair, and resistance to apoptosis following a stroke (Lv et al., 2022a).

The NP-medicated activation of CREB has yielded promising results in the enhancement of neuronal survival and synaptic recovery, outcomes that are often unattainable with conventional therapies (Wu and Angelov, 2023). These advancements indicate the superior neuroprotective potential of NPs in the treatment of IS. The PI3K/AKT/CREB signaling pathway plays a crucial role in neuroprotection, regulating neurogenesis, cell survival, and the suppression of apoptosis; these are key processes for mitigating the damage caused by IS (Zhang et al., 2007; Chen et al., 2022). The activation of CREB promotes hippocampal neurogenesis, and the inhibition of PDE4 enhances cAMP-CREB signaling, thus representing a promising therapeutic strategy (Jin et al., 1999; Zhu et al., 2004).

Current research in nanotechnology is focusing on the PI3K/AKT pathway to exert neuroprotective effects by activating AKT and regulatory factors such as CREB and mTOR to enhance cellular resilience (Liu and Graybiel, 1997; Rafalski and Brunet, 2011; King et al., 2015; Li et al., 2018a). The response of CREB to oxidative stress, particularly in terms of phosphorylation at Ser-133, has been shown to improve cognitive resilience and promote neuronal proliferation (Schmid et al., 1999; España et al., 2010; Rehman et al., 2019). Emerging NP-based therapies are designed to activate the PI3K/AKT/CREB signaling pathway following a stroke. For instance, liposomal NPs carrying neurotrophic agents such as GDNF promote neuronal survival, while curcumin-loaded nanostructured lipid carriers (NLCs) protect brain tissue (Bahlakeh et al., 2021). Lipid-based NPs, such as liposomes, enhance BBB penetration and deliver factors such as BDNF, thereby activating the PI3K/AKT/CREB pathway to support synaptic plasticity and functional recovery (Wu and Angelova, 2023). Polymeric NPs can release drugs in response to ischemic triggers, such as pH changes and oxidative stress, thus enabling localized activation of the PI3K/AKT signaling pathway (Karam et al., 2022). Research on polymeric NPs for cortical repair has demonstrated enhanced stem cell proliferation and neuroprotection via the PI3K pathway (Ebrahimi-Barough et al., 2017). Furthermore, curcumin-loaded exosomes and advanced co-delivery NPs have been shown to reduce oxidative stress and mitochondrial dysfunction; these are key contributors to ischemic brain injury (He et al., 2020b). Inorganic NPs, such as SiO_2_, are known to activate the PI3K/AKT/CREB pathway; subsequently, CREB regulates Bcl-2 transcription to protect against apoptosis and support neuroprotection (Creson et al., 2009; Rauch et al., 2013; Nazemidashtarjani et al., 2020; Francia et al., 2022). This integrated understanding of the manner by which NPs can modulate the PI3K/AKT/CREB signaling pathway opens new therapeutic avenues for IS and broader neurological applications.

**Figure 5:**
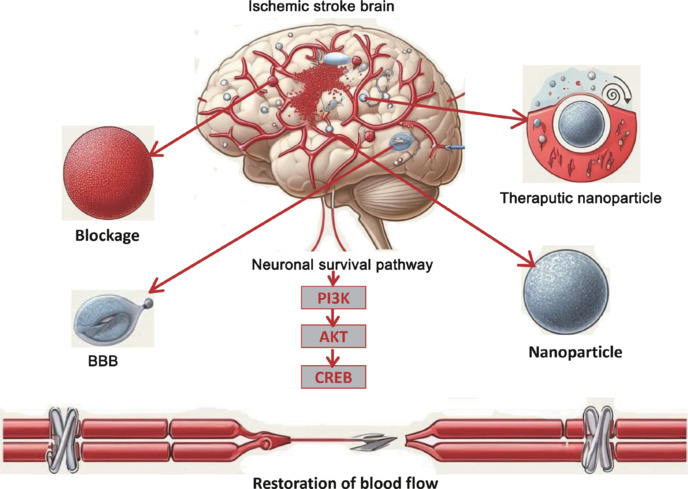
Nanoparticles could enhance the treatment of ischemic stroke: Improving neural protection and promoting blood flow recovery. This graphic illustrates the role of nanoparticles in the treatment of ischemic stroke by focusing on the PI3K/AKT/CREB pathway, which enhances neuronal survival. We highlight key stroke-related mechanisms, including interactions with the BBB, vascular blockage, and the application of therapeutic nanoparticles to improve neuroprotection and restore injured brain tissue. The lower section of the graphic emphasizes the potential benefits of nanoparticle-based therapies in stroke recovery, which shows a revival of blood flow. AKT: Protein kinase B; BBB: blood–brain barrier; CREB: cAMP response element-binding protein; PI3K: phosphatidylinositol 3-kinase.

NPs provide a transformative approach for the treatment of stroke by targeting the PI3K/AKT/CREB pathway to enhance neuronal survival and promote synaptic repair. NPs can be engineered for specific targeting, thereby improving efficacy and safety in post-stroke interventions (Ruscu and Cercel, 2023). Advances in NP engineering, particularly via the use of responsive polymers, gold nanorods, and liposomal NPs, have successfully overcome barriers such as the BBB and reduced neuroinflammation (He et al., 2021b). Gold nanorods and liposomal NPs enhance biocompatibility and minimize immune responses, thus providing sustained, localized effects at the injury site (Raju and Abuwatfa, 2023). The NP-mediated activation of CREB promotes neuronal survival and facilitates synaptic recovery, thus surpassing the efficacy of conventional therapies (Wu and Angelova, 2023). These advancements highlight the superior neuroprotective potential of NPs for the treatment of IS. **Figure 5** illustrates the role of NPs in the treatment of IS by targeting the PI3K/AKT/CREB pathway to promote neuronal survival and neuroprotection. **Figure 5** emphasizes interactions with the BBB, vascular obstruction, and the ability of therapeutic NPs to enhance brain recovery, thus indicating the restorative potential of NP-based therapies via the restoration of blood flow.

### Nanoparticles-based modulation of the PI3K/Akt/CREB pathway: A targeted strategy for neuroprotection in ischemic stroke

Following IS, the PI3K/Akt/CREB pathway plays a crucial role in mediating neuroprotection via numerous components, such as GluN2A, TRPC6, NGF/TrkA, and BDNF/TrkB, while also promoting neurodegeneration via GluN2B, TRPM7, and TRPV1, thus indicating potential targets for intervention. Early research identified the NMDA receptor as a key regulator of glutamate-mediated neurodegeneration in IS. NMDA receptors are composed of two GluN1 subunits and two GluN2 subunits (which bind glutamate). GluN2A, primarily expressed at synapses, enhances cell survival by stimulating prosurvival pathways, including PI3K/Akt and CREB. In contrast, GluN2B, which is predominantly found in extrasynaptic locations, triggers pro-death pathways, such as the activation of neuronal nitric oxide synthase (Wu and Tymianski, 2018).

GluN2B is the primary active NMDA receptor during IS and contributes to cerebral ischemia/reperfusion (I/R) injury. Therefore, a potential neuroprotective strategy could involve specifically blocking GluN2B or its downstream pro-death pathways. Non-glutamate-dependent cation channels, such as transient receptor potential channels and acid-sensing ion channels, also play a role in regulating Ca^2+^ influx (Sun, 2017). Of the six TRP channel subgroups, TRPC6, TRPM7, and TRPV1 have been extensively studied in patients with IS. TRPM7 and TRPV1 promote neuronal death by increasing intracellular levels of Ca^2+^, whereas TRPC6 supports neuronal survival by activating the CREB and CaMK (calmodulin-dependent protein kinase) signaling pathways (Cao et al., 2017). Akt has been shown to promote the phosphorylation of Bad, an inhibitor of Bcl-2. Once phosphorylated, Bad dissociates from Bcl-2, thus allowing Bcl-2 to bind to the mitochondria, thereby inhibiting activation of the mitochondrial permeability transition pore and preventing the release of cytochrome c (Franke et al., 2003). Most anti-apoptotic drugs in preclinical models of IS target the PI3K/Akt signaling pathway due to its critical role in cerebral I/R-induced apoptosis. For example, the neuroprotective effects of puerarin and silibinin (silybin) have been linked to the activation of the PI3K/Akt signaling pathway (Tao et al., 2017). Neurotrophins regulate neuronal survival, development, and recovery. In IS, NGF binds to TrkA, activating the Erk/CREB pathway, while BDNF, the most abundant neurotrophin in the brain, binds to TrkB, triggering PI3K/Akt and MAPK signaling for neuroprotection (Houlton et al., 2019). The expression of both NGF/TrkA and BDNF/TrkB increases post-stroke as part of a self-rescue mechanism.

Neuroprotective targeted mechanisms aim to shield neurons from damage caused by conditions such as IS, neurodegenerative diseases, and traumatic brain injury through specific therapeutic strategies. These mechanisms reduce oxidative stress, mitigate inflammation, maintain mitochondrial function, and promote neurotrophic signaling; these are complex processes that collectively preserve neuronal health. These processes act in a coordinated manner to prevent apoptosis, inhibit pro-inflammatory cytokines, reduce protein misfolding, regulate calcium homeostasis, and neutralize free radicals. Key strategies include enhancing redox balance, improving mitochondrial bioenergetics, increasing the production of neurotrophic factors, avoiding excitotoxicity, and optimizing the control of protein quality. This comprehensive defense strategy supports long-term cerebral resilience, enabling neurons to resist damage from oxidative stress, inflammation, metabolic disturbances, and neurodegeneration.

**Figure 6:**
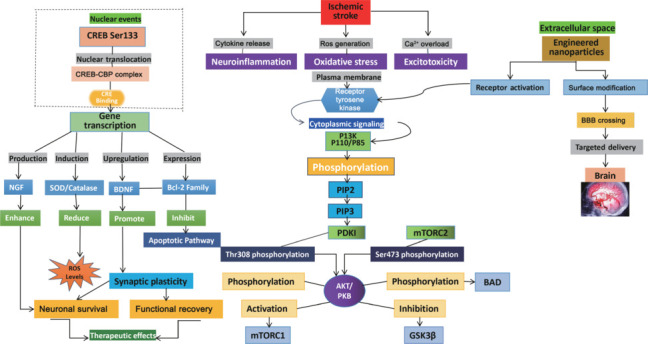
Key molecular processes in ischemic stroke: neuroinflammation, oxidative stress, and excitotoxicity contributing to neuronal damage. These processes engage critical signaling pathways, such as the PI3K/AKT/CREB pathway, which promote neuronal survival and repair. The PI3K/AKT pathway prevents cell death by activating protective signals, while CREB regulates the expression of neuroprotective genes. We also illustrate how engineered nanoparticles facilitate the targeted delivery of therapeutic agents to ischemic brain regions. These nanoparticles are designed to cross the BBB and release drugs in a controlled manner, enhancing the efficacy of stroke treatment while minimizing side effects. AKT/PKB: Protein kinase B; BAD: BCL2 associated agonist of cell death; BBB: blood–brain barrier; BDNF: brain-derived neurotrophic factor; Ca^2+^: calcium ion; CREB: cAMP response element-binding protein; GSK3β: glycogen synthase kinase 3 beta; mTORC1: mechanistic target of rapamycin complex 1; mTORC2: mechanistic target of rapamycin complex 2; NGF: nerve growth factor; PDK1: 3-phosphoinositide-dependent protein kinase 1; PI3K: phosphatidylinositol 3-kinase; PIP2: phospha tidylinositol 4,5-bisphosphate; PIP3: phosphatidylinositol (3,4,5)-trisphosphate; ROS: reactive oxygen species; SOD: superoxide dismutase.

NPs can be engineered to target specific pathological processes, offering precise therapeutic effects. Understanding the complex mechanisms underlying IS, such as oxidative stress and neuroinflammation, highlights the need for targeted interventions. NPs have been designed to interact directly with stroke-related molecular pathways, including those involved in oxidative stress and inflammation, thus enabling targeted delivery and enhancing neuroprotection (Kumar et al., 2021). Following stroke, ischemic injury disrupts critical cellular signaling pathways, such as the PI3K/AKT/CREB axis, which is essential for neuronal survival, repair, and neuroprotection (**Figure 6**). When these pathways are impaired, the cellular response to injury weakens, leading to increased oxidative stress, inflammation, and apoptosis. This dysfunction exacerbates damage in ischemic regions of the brain, resulting in irreversible neuronal loss and hindering recovery (Gu et al., 2022). The long-term consequences for stroke survivors are exacerbated when appropriate signaling through these pathways is not restored, thus delaying healing processes and diminishing the capacity of the brain to recover from ischemic damage. Excitotoxicity due to calcium overload, oxidative stress from excessive ROS, and neuroinflammation are the three primary causes of cellular damage in the pathological context of IS (Pawluk and Tafelska-Kaczmarek, 2024).

To reverse these effects, it is essential that the PI3K/AKT pathway is activated. Upon activation, PI3K recruits and activates PDK1 by phosphorylating PIP2 to PIP3. This leads to the phosphorylation of AKT at Thr308, while mTORC2 phosphorylation at Ser473 provides additional stability and enhances the activity of AKT (Jazirehi et al., 2012). By influencing downstream effectors, such as GSK3β and pro-apoptotic proteins such as BAD, activated AKT reduces neuronal death and promotes synaptic plasticity (Pungsrinont et al., 2021). Furthermore, AKT accelerates the nuclear translocation of CREB by increasing its phosphorylation at Ser133. According to Moore et al. (1996), once in the nucleus, CREB binds to CBP and stimulates the production of antioxidant enzymes and neuroprotective genes, including Bdnf and Bcl-2. NPs differ from conventional therapeutic methods due to their multimodal activity. Their surface engineering optimizes charge properties for cellular absorption, including PEGylation for extended circulation and specialized targeting ligands for enhanced cellular recognition (Gu and Minko, 2024). Once inside target neurons, NPs enhance endogenous repair processes, modulate inflammatory responses, and provide antioxidants, collectively exerting impact on several protective mechanisms. According to Liu et al. (2017), these particles utilize pH-sensitive bonding to release drugs in affected regions in a controlled manner in response to the ischemic microenvironment. This comprehensive strategy provides long-lasting neuroprotection through direct benefits, such as scavenging free radicals, as well as indirect effects, including the enhancement of cellular repair processes and the modulation of inflammatory cell infiltration (Zong et al., 2024).

### Nanoparticles-mediated modulation of ischemic stroke in experimental models via the PI3K/AKT/CREB signaling pathway

Recent studies suggest that NP-based formulations can enhance drug delivery across the BBB and modulate key signaling pathways, such as PI3K/AKT/CREB, which are crucial for neuronal survival and synaptic plasticity. However, further validation from *in vivo* models is needed to confirm their efficacy for the treatment of IS (Dong et al., 2020; Liu et al., 2023c). The PI3K/AKT pathway plays a vital role in promoting neuronal survival by suppressing apoptosis (Morrison et al., 2002; Babu et al., 2017). Effective synaptic connections and targeted drug delivery are essential, as impaired synapse formation can lead to neuronal death. Researchers are investigating various NPs that can target the PI3K/AKT/CREB axis, including polymeric NPs (such as PLGA), CXCR4-targeted NPs for focused delivery, and mesenchymal stem cell-derived exosomes (MSC-exosomes) with therapeutic potential (Bernardo-Castro et al., 2021). Magnetic Fe_3_O_4_ NPs and inorganic NPs (e.g., silica and iron oxide) show promise for targeted delivery and diagnostic applications (Dong et al., 2020). Furthermore, lipid-peptide nano-assemblies and curcumin-loaded liquid-LNPs modulate pathways such as the BDNF/TrkB and CREB pathways, thereby enhancing neuronal resilience in models of stroke (Morrison et al., 2002; Rakotoarisoa et al., 2022). Targeted micelles and other nanocarriers also activate pathways such as PI3K/AKT pathway, offering potential for IS treatment (Rakotoarisoa et al., 2022). In mouse models of middle cerebral artery occlusion, Ngb nanocapsules were found to upregulate proteins such as ATXN2L and NTRK2, a BDNF receptor associated with neuroplasticity and mitochondrial remodeling (Lin et al., 2019; Peinado et al., 2021).

In rat models of chronic cerebral hypoperfusion (CCH), activation of the PI3K/Akt/CREB pathway has been shown to protect memory. However, the use of nanomaterials poses potential cytotoxic and genotoxic risks (Vecchio et al., 2014; Fard et al., 2015). Encapsulated basic fibroblast growth factoractivates the PI3K/Akt pathway to promote neuron viability and synaptic plasticity (Chrissouliet al., 2010; Landowski et al., 2020). Conversely, the suppression of VEGF-A via the PI3K/Akt pathway can inhibit neuronal growth (Ortega et al., 2021). Sesamol-loaded nanostructured lipid carriersand liposome-based plasminogen activators show promise for stroke therapy by reducing side effects and enhancing stability (Koudelka et al., 2016; Vaidya et al., 2016). Adipose-derived stem cell exosomesinhibit IGFBP5 which can enhance cell survival via the PI3K/Akt pathway in models of subarachnoid hemorrhage (Bilal et al., 2020; Wang et al., 2022b). BDNF prevents neuronal damage by phosphorylating CREB and modulating the PI3K/Akt and ERK pathways (Xu et al., 2007; Xia et al., 2010). Furthermore, curcumin-loaded nanostructured lipid carriers and encapsulated basic fibroblast growth factor have demonstrated neuroprotective effects by activating the PI3K/Akt/CREB pathway and reducing neurological damage (Zhao et al., 2016; Qin et al., 2022). However, concerns relating to neurotoxicity have been raised with regards to silver nanoparticles, which induce autophagy by activating AKT/mTOR (Blanco et al., 2018).

Innovative approaches, such as H_2_O_2_-responsive polymer NPs and gold NPs, which target CREB and BDNF, show potential for neuroprotection (Gao et al., 2017; Strużyńska and Skalska, 2018). However, silica NPs have been found to induce neurotoxicity via ROS production and modulation of the PI3K/Akt/CREB/Bcl-2 signaling pathway (Napierska et al., 2010). Tailored polymeric NPs that deliver siRNA and 3D nano-scaffolds have been shown to improve cell viability and reduce the proliferation of astrocytes under ischemic conditions (Boni et al., 2020; Zhou et al., 2022). These advancements highlight the potential of NPs to enhance the efficacy and safety of treatment for IS; however, further research is needed to optimize delivery systems and evaluate their long-term safety.

## Clinical Translation

One major concern is safety, particularly regarding the long-term consequences and potential off-target effects on immunological responses, which are still poorly understood. In addition, the manufacturing processes for complex therapeutic compounds, such as exhibitors, berberine, inhibitors, and the compound FORKS, need to be improved to enable cost-effective scaling to meet clinical demands (Sun et al., 2024). Regulatory challenges further complicate the process, as thorough preclinical research and human trials are required to demonstrate safety and efficacy. Despite these obstacles, promising models suggest that these treatments hold significant potential.

**Figure 7:**
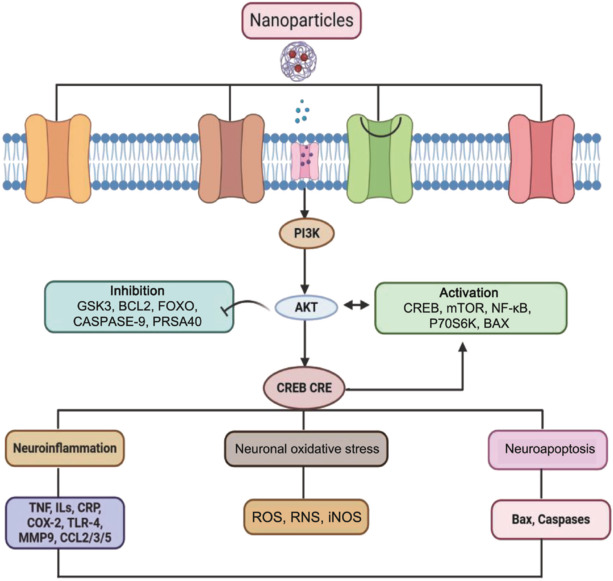
Targeting the PI3K/AKT/CREB pathway with nanoparticles for stroke therapy. Nanoparticles target the PI3K/AKT/CREB signaling pathway, which is involved in stroke, for therapeutic applications. AKT activation inhibits CREB. AKT: Protein kinase B; BCL2: B-cell lymphoma 2; CCL2/3/5: chemokine (C-C motif) ligand 2/3/5; COX-2: cyclooxygenase-2; CRE: cAMP response element; CREB: cAMP response element-binding protein; CRP: C-reactive protein; FOXO: forkhead box O transcription factor; GSK3: glycogen synthase kinase 3; ILs: interleukins; iNOS: inducible nitric oxide synthase; MMP9: matrix metallopeptidase 9; mTOR: mechanistic target of rapamycin; NF-κB: nuclear factor kappa-light-chain-enhancer of activated B cells; P7056K: ribosomal protein S6 kinase beta-1; PI3K: phosphatidylinositol 3-kinase; PRSA40: proline-rich acidic protein 40; RNS: reactive nitrogen species; ROS: reactive oxygen species; TLR-4: toll-like receptor 4; TNF: tumor necrosis factor.

Clinical studies of monoclonal antibodies targeting leukocyte-endothelial interactions have shown promise for the reduction of inflammation caused by stroke, thus suggesting potential benefits for therapies based on CAMS (Milošević et al., 2022). Similarly, NP-based treatments for IS offer significant potential but face substantial challenges. The development of biodegradable, non-immunogenic NPs is essential due to concerns relating to toxicity, including long-term biocompatibility and clearance (Bernardo-Castro and Albino, 2021). Scalability is another critical issue; production techniques such as three-dimensional (3D) printing and microfluidics must be advanced to enable large-scale manufacturing without compromising quality (Luo et al., 2023). The complexity of regulatory approval processes further indicates the need for collaborative efforts to expedite these pathways, as thorough safety and pharmacokinetic assessments are still required (Franco et al., 2023). Overcoming these obstacles is crucial to bring these innovative treatments closer to clinical application. Successful clinical translation will depend on a stepwise approach, which includes the conduction of multicenter clinical studies, utilizing bioinformatics to develop individualized treatment plans, and fostering partnerships to streamline manufacturing and delivery. Furthermore, addressing ethical and financial considerations will be vital to ensure that these treatments are relevant and effective in real-world settings. Recent studies have increasingly emphasized the critical role of the PI3K/Akt/CREB signaling pathway in the CNS and its dysfunction (**Figure 7**).

### Drug delivery

NPs for drug delivery can be synthesized from either synthetic or organic materials, including micelles, liposomes, polymers, and metallic particles. The specific properties of NPs, such as polarity, surface receptors, and lipophilicity, can be tailored to enhance drug delivery via encapsulation or conjugation. The size of NPs (ranging from 1 to 300 nm) significantly influences drug release, with smaller NPs allowing for faster release, while larger NPs may hinder efficient delivery. Controlled release is essential to prevent premature metabolism and clearance. NPs can also be modified with biological components, such as peptides and antibodies, which enhance their efficacy as drug carriers (Panagious and Saha, 2015). Polymeric micelles, particularly those made from Food and Drug Administration (FDA)-approved Poly(lactic-co-glycolic acid) PLGA, are widely used for the delivery of hydrophobic drugs due to their biodegradability and biocompatibility (Sahoo et al., 2023). Modified polymeric NPs offer targeted, ligand-based release with improved biocompatibility and biodegradability, thus revolutionizing drug delivery by enhancing precision, bioavailability, and efficacy while minimizing toxicity and off-target effects. Their ability to cross biological barriers, such as the BBB, makes them vital for treating complex neurological disorders (Villanueva-Flores et al., 2020).

Stimuli-responsive nanocarriers enable precise drug delivery and controlled release, reducing adverse effects while maintaining the efficacy of bioactive compounds. Advanced drug delivery systems target site-specific factors such as ROS and pH, thereby improving thrombolysis and neuroprotection (Zhang et al., 2022a). NPs designed to breach the BBB can deliver therapeutics directly to ischemic regions, thus enhancing bioavailability and minimizing systemic side effects. By reducing ROS, inflammation, and apoptosis, these NPs promote neuronal survival and functional recovery via the PI3K/AKT/CREB pathway (Herdiana et al., 2023). Recent advances in NP engineering have optimized BBB penetration via size-dependent transcytosis and receptor-mediated transport, thus initiating neuroprotective cascades via PI3K/AKT/CREB signaling. This process enhances the expression of survival genes, such as BDNF and BCL-2 (Bahar et al., 2023). Various NPs, including PLGA, liposomes, and metallic or biomimetic types, facilitate controlled drug release, improving therapeutic outcomes while reducing the required dosage. However, challenges related to scalability, stability, and regulatory approval persist (Hassanpour and Kim, 2020). For example, liposomal NPs specifically target ischemic regions of the brain, controlling neuroinflammation and enhancing drug bioavailability (Zhang et al., 2021a). Polymeric micelle nanoparticles, formed from amphiphilic polymers, improve key therapeutic properties such as a longer half-life and reduced immunogenicity, thus facilitating BBB penetration and targeted delivery (Cagel et al., 2017). Dendrimers, such as PAMAM, show promise for drug and gene delivery in ischemic stroke due to their low cytotoxicity and high transfection efficiency; however, further research is needed to confirm their long-term efficacy (Huang et al., 2022). Extracellular vesicles and stem cell-derived exosomes provide biocompatible and low-toxicity options for targeted drug delivery. However, challenges such as production costs and donor variability hinder their clinical translation (Wiklander et al., 2015; Tian et al., 2018).

Targeted ligands, such as those binding to integrins or microglia, enhance NP delivery to brain lesions, while neutrophil-targeting peptides such as Ac-PGP improve the targeting of ischemic regions (Zhang et al., 2017). NPs also protect unstable or toxic cargo, allowing for the efficient delivery of small molecules such as glyburide and NA1 to the brain (Zhang et al., 2022b). Conjugated phospholipid NPs loaded with tPA and Akt-stimulating peptides enhance angiogenesis and neuroprotection in animal models (Liao et al., 2020; Annu et al., 2022). Furthermore, NPs targeting LCP1 in macrophages mitigate ischemic injury by modulating immune signaling (Liu et al., 2023a). Sequential infusions of growth factors, such as erythropoietin and epidermal growth factor, stimulate endogenous neuronal stem cells, effectively bypassing the BBB (Wang et al., 2013). Advances in MRI-guided NP delivery and plasmonic gold NPs activated by near-infrared light can further enhance therapeutic outcomes (Odion et al., 2021; Li et al., 2023). Despite challenges related to standardization and scalability, NPs hold immense potential for the treatment of stroke, particularly via the PI3K/AKT/CREB pathway. However, further research is needed to optimize their design, delivery, and safety (Qin et al., 2022). The integration of artificial intelligence, biosensors, and optimized manufacturing processes continues to advance nanomedicine, offering precise and effective treatments for neurological disorders.

### Clot-reflux agents

The interaction between clot-reflex agents and PI3K/AKT/CREB-targeting NPs for the treatment of stroke remains unclear. However, therapeutic modulators, such as BDNF and insulin-like growth factor 1, can activate this pathway to promote neuroprotection. NPs enhance stroke recovery by improving drug delivery across the BBB, offering a revolutionary approach for the treatment of stroke via precise molecular design. These advanced carriers target specific cellular mechanisms, mitigating stroke damage and promoting neural recovery with unprecedented precision (Alkaff et al., 2020).

Innovative nanocarriers, including liposomes, polymeric micelles, dendrimers, and extracellular vesicles, enable targeted drug delivery across complex biological barriers, thereby transforming stroke therapeutics. Thrombosis, caused by an imbalance between clotting and fibrinolysis, poses significant health risks and necessitates targeted treatments. Traditional thrombolytic drugs face some major challenges, including short half-lives, the risk of bleeding, and adverse effects, thus highlighting the need for improved therapies. Nanotechnology addresses these issues by providing advanced carriers, such as liposomes and magnetic NPs, which can enhance drug stability, targeting, and safety (Shen et al., 2021).

Thrombosis is triggered by factors such as collagen, thrombin, and tissue factor, leading to platelet activation and the polymerization of fibrin. Nanocarriers engineered with targeting moieties, such as peptides or antibodies, can improve thrombolysis by delivering plasminogen activators that dissolve clots, specifically targeting biomarkers such as P-selectin and integrin GPIIb/IIIa (Guan and Dou, 2021). Activated platelet-sensitive nanocarriers enable the targeted delivery of thrombolytic drugs such as tPA to enhance clot lysis while minimizing systemic side effects. For instance, cRGD-coated, PEGylated liposomes selectively target the αIIbβ3 integrin, thus improving thrombolytic efficacy (Huang et al., 2019). Fucoidan-functionalized polysaccharide NPs target P-selectin on active platelets, enhancing the deposition of recombinanttissue plasminogen activator (rtPA) at thrombus sites. However, P-selectin-based targeting is most effective when combined with other strategies, as it is also utilized for cancer therapy (Singh et al., 2016). Liposomes are favored for their biocompatibility and low immunogenicity, and can be surface-modified for thrombus targeting. Advanced formulations, such as echogenic liposomes and magnetic NPs, further improve clot penetration and targeting when combined with ultrasound or magnetic fields (Ma et al., 2021).

Recently, there have been significant advances in the application of biomimetic and biogenetic nanomaterials for thrombolytic therapy. Magnetic-guided targeting using magnetic NPs and external magnetic fields allows for precise thrombolysis. Coated magnetic NPs not only reduce toxicity but also improve the efficiency of clot lysis at lower doses of rtPA (Liu et al., 2019). Biomimetic approaches, such as red blood cell (RBC)-based delivery systems, utilize the long circulation time of RBCs for targeted rtPA delivery. For example, RBC/tPA complexes have been shown to effectively lyse cerebral thromboemboli, although their large size limits tissue penetration (Danielyan et al., 2008). Shear-activated nanotherapeutics release rtPA under high shear stress, enabling targeted delivery in stenotic vessels (Absar et al., 2014). Bio-responsive strategies, such as albumin-camouflaged nanocarriers, protect rtPA during circulation but release it at clot sites, enhancing thrombolytic efficacy while minimizing side effects (Absar et al., 2014). However, rtPA can increase ROS, leading to secondary ischemia/reperfusion injury (Mei et al., 2019). NPs address this issue by targeting the sites of thrombi, extending drug circulation, and reducing adverse effects. Emerging techniques, including dual-targeting and theranostics, combine imaging tools with rtPA delivery, although further optimization is still necessary. Despite their potential, the complexity of *in vivo* models and the need for extensive research hinder the clinical translation of bioinspired nanomaterials.

## Nanoparticles for Stroke Therapy: Food and Drug Administration–Approved Drugs, Clinical Protocols, and Current Clinical Studies

Most FDA-approved NPs are liposomal or polymeric, and newer types are expected to gain traction (Bobo et al., 2016). The use of NPs for mRNA delivery is also increasing (Anselmo and Mitragotri, 2019). Regulatory bodies, such as the European Medicines Agency and the FDA, have issued guidelines to support the development of nanomedicine to address both scientific and regulatory challenges (FDA, 2017; Çapan et al., 2021). While drugs such as adenosine show potential for reducing inflammation and apoptosis, their short half-life and poor BBB permeability limit their use for the treatment of cerebral ischemia-reperfusion injury (Chen and Gao, 2017). However, ongoing research is investigating the use of NPs to improve drug delivery for stroke treatment. tPA, which is FDA-approved for ischemic stroke, dissolves blood clots by targeting networks of fibrin (Wang et al., 2021c). Polymer-based NPs, such as SHp-RBC, respond to ROS to enhance drug bioavailability and targeting efficiency (Lv et al., 2018). Engineered “nano-platelets” (tP-NP-rtPA/ZL006e) offer sequential delivery for thrombolysis and neuroprotection, although the potential risks of hepatotoxicity require further evaluation (Yang et al., 2023). Furthermore, NBP-CeO₂ NPs combine antioxidant and neurovascular healing properties, suggesting a novel approach for the treatment of IS (Li et al., 2022b).

Liposomes and poly(lactic-co-glycolic acid)-based NPs show great promise for IS therapy due to their ability to target the BBB and deliver both neuroprotective and antioxidant drugs (Sarmah et al., 2021; Zhang and Huang, 2024). BDP-4/Cur-CL NPs allow for the real-time monitoring of peroxynitrite (ONOO^–^) and facilitate tailored curcumin therapy, thus contributing to both diagnosis and recovery (Zhang and Wang, 2023). Iron oxide (Fe_3_O_4_) NPs, which are FDA-approved for MRI and the treatment of iron deficiency, exhibit enzyme-like properties that can help to reduce oxidative damage and neuronal death in IS (Yan et al., 2020). Prussian blue NPs, approved for the treatment of heavy metal poisoning, act as ROS scavengers, providing neuroprotection by reducing inflammation and apoptosis (Zhang et al., 2019a). Recent advancements include magnetoelectric NPs (Me-NPs) and CeO_2_@ZIF-8, which offer antioxidative and anti-inflammatory benefits by eliminating ROS (He et al., 2020a). Mesoporous silica NPs, known for their large surface area and controlled drug release capabilities, also show potential for the treatment of stroke, although further research is needed (Cha et al., 2018). Gold nanoparticles (AuNPs), modified with thiol or amine groups, enhance the targeting of thrombolytic drugs and imaging. For instance, Au@MSNs improve thrombolysis in IS by exerting photothermal effects (Wang et al., 2017b). Dl-3-N-butylphthalide (NBP), a novel drug approved by the FDA for the treatment of IS, enhances neuroprotection by improving microcirculation, reducing inflammation, and by promoting repair processes (Li et al., 2022b). These advancements highlight the potential of NPs for stroke therapy, although challenges in clinical translation remain. Key developments in targeted drug delivery, neuroprotection, antioxidant properties, and BBB restoration are shown in **Table 2**. These innovations will help to enhance the outcomes of stroke treatments by strengthening precision medicine approaches.

**Table 2:** Transformative nanoparticle technologies: Navigating clinical advances in stroke therapy

NP type	Mechanism	Efficacy	Milestone	Reference
Tissue plasminogen activator NPs	Fibrin network dissolution	Enhanced drug delivery	Improved bioavailability targeting	Wang et al., 2021b
Nano-platelets	Sequential drug delivery	Targeted thrombolysis	Enhancing neuroprotection	Yang et al., 2023
CeO_2_ NPs	Multimodal imaging platform	Precision medicine approach	NIR-triggered drug release	He and Huang, 2020
Lf-targeted lipid carriers	BBB targeting	Neuroprotective drug transport	Extended drug circulation time	Zhao et al., 2018
PEG/cRGD liposomes	Neuroinflammation	Functional recovery enhancement	Reduced infarct volume	Wang et al., 2020
Edaravone-loaded cerium NPs	ROS scavenging	Neuronal protection	Improved brain penetration	Bao et al., 2018
Manganese-based PNzyme/MnO_2_ NPs	Antioxidant	Ischemia/reperfusion mitigation	Oxidative stress management	Wang et al., 2024
γ-Fe_2_O_3_, Fe_3_O_4_, magnetic-iron oxide NPs	Targeted cellular interaction	BBB repair mechanism	Magnetic-guided drug delivery	Shen et al., 2018
NO magnetic NPs. Ca-MOF@miR-124 NPs	Arterial recanalization	Diagnostic imaging integration	Theranostic strategy	Li et al., 2020
Hydrogels	Antioxidant, BBB protection	Management of hemorrhagic cerebral edema	Dual-action therapy	Liu et al., 2023
Hemostatic hydrogels	Control of cerebral edema	Hemostatic management	Treatment of hemorrhagic stroke	Rotaru-Zăvăleanu et al., 2024
EDV/Cur/NapFFYhydrogel	Synergistic neuroprotection	Promotion of cerebral plasticity recovery	Using supramolecular peptide hydrogels	Jia et al., 2023
cRGD/TPP@Resmicelles	Reduction of oxidative stress	Mitigation of neurological damage	Mitochondrial-targeted therapy	Wang et al., 2023
miRNA-loaded calcium NPs	Activation of neural stem cells	Neurological recovery	Neuroregeneration	Yan et al., 2020

This table elaborates the mechanisms, therapeutic potential, significant advancements, including enhanced drug delivery neuroprotection, of the different NPs utilized in stroke treatment. It also cites research showing how well they work to address stroke–related issues such as BBB crossing, ischemia-reperfusion injury, and thrombolysis. From single-target to multidisciplinary methods, nanoparticle-based stroke treatments have developed, with several platforms tackling important pathogenic mechanisms: Although Lf-targeted carriers including PEG/cRGD liposomes that target BBB penetrating neuroinflammation, tissue plasminogen activator NPs nanoplatelets improve drug delivery dissolution of clots. Cerium, manganese-based NPs are used to regulate oxidative stress, magnetic guiding devices are used for targeted administration. Cutting-edge hydrogel systems and new technologies, such as miRNA-loaded calcium NPs (cRGD/TPP@Res micelles), further illustrate the field's shift toward precision medicine with enhanced therapeutic efficacy. BBB: Blood–brain barrier; CeO_2_: ceriumdioxide; Fe_3_O_4_: magnetic iron oxide; Lf: lactoferrin; MnO_2_: manganese dioxide; NO: nitric oxide; NPs: nanoparticles; PEG/cRGD: Polyethylene Glycol conjugated with cyclic Arginyl–Glycyl–Aspartic acid; PNzyme: peroxidase-mimickling nanozyme; ROS: reactive oxygen species.

## Limitations

NPs targeting the PI3K/AKT/CREB pathway show promise for the treatment of IS but face significant challenges. The broad cellular roles of this pathway, along with the unclear mechanisms of NP modulation, have raised concerns relating to potential off-target effects. Translating preclinical findings into clinical applications is hindered by interspecies variations, limited clinical trials, and safety issues, such as NP toxicity and long-term biocompatibility. The multifactorial nature of IS, which involves excitotoxicity, inflammation, and oxidative stress, further complicates the development of appropriate therpaeutics. Current strategies often focus on acute neuroprotection, overlooking the importance of long-term rehabilitation and functional outcomes. This highlights the need for comprehensive and clinically validated approaches. To advance NP-based therapies, researchers must develop advanced surface modifications and delivery systems that interact reliably with neuronal cells, regulate drug release, and promote functional recovery. Bridging the gap between research and clinical use requires innovative solutions that balance therapeutic potential, safety, and regulatory compliance. Key challenges include optimizing BBB penetration, minimizing systemic toxicity, achieving precise cellular targeting, and controlling oxidative damage while ensuring long-term efficacy. Furthermore, regulatory approval remains a hurdle, demanding rigorous safety assessments and pharmacokinetic studies.

NPs hold immense potential to overcome the limitations of conventional stroke therapies via targeted delivery and multimodal action. Future research should prioritize the development of biocompatible and biodegradable systems to reduce toxicity and enhance safety. Scalable production techniques, such as microfluidics and AI-driven design, are essential for the widespread adoption of these therapies. Rigorous clinical trials are necessary to validate efficacy and address regulatory barriers. Beyond stroke, the adaptability of NPs makes them promising candidates for treating other neurological disorders. With continued innovation and collaboration, NP-based therapies could revolutionize the management of stroke and significantly improve patient outcomes.

Nanotechnology could transform the treatment of stroke by enabling precise drug delivery and targeted neural repair. NPs, via advanced molecular targeting and the integration of signaling pathways, offer new opportunities for neuronal regeneration. The convergence of clinical neuroscience, computational design, and molecular engineering points toward personalized, mechanism-driven stroke therapies. While NP-based treatments show great promise, overcoming challenges such as toxicity, biocompatibility, and design optimization is crucial. Combining multimodal therapies with personalized approaches could enhance recovery and redefine stroke care.

## Discussion

### Future perspectives

NP therapies targeting the PI3K/AKT/CREB pathway represent a promising advancement for the treatment of IS. These therapies enable precise modulation of neuroprotective pathways and enhance drug delivery across the BBB. Future research should concentrate on developing dual-trigger designs that respond to both oxidative stress and specific cellular enzymes in ischemic tissue, thereby improving targeting accuracy. In addition, creating biodegradable coatings and enhancing BBB penetration will further support biocompatibility and safety.

Combined approaches, such as integrating NPs with neuroprotective drugs or gene therapies, could further enhance efficacy by addressing both primary and secondary injuries. Artificial intelligence (AI)–driven NP design can optimize properties for improved BBB penetration and targeting precision, accelerating development and reducing costs. In addition to stroke, these therapies hold potential for neurodegenerative diseases such as Alzheimer’s disease and Parkinson’s disease, targeting oxidative stress and inflammation. Theranostic NPs, which combine therapy and diagnostics, enable the real-time monitoring of drug delivery, while their biodegradable designs ensure safety and biocompatibility. Innovations in scalable production and rigorous clinical trials are essential for successful clinical translation, potentially revolutionizing neurotherapeutics.

Emerging NPs are improving the treatment of stroke by precision and multifunctionality. Dual-trigger systems that respond to pH changes and oxidative stress can release targeted antioxidants and anti-inflammatory agents, such as curcumin, in ischemic conditions. Gold nanorods combine imaging and therapy by using near-infrared light to reduce damage (Zhan et al., 2023). AI-enhanced NP design offers smarter therapeutic options, improving drug delivery and targeting for both ischemic and hemorrhagic strokes.

### Conclusions

The use of NP-based therapies targeting the PI3K/AKT/CREB pathway represents a revolutionary advancement in the management of IS. These approaches address critical challenges in the treatment of IS, including neuroprotection, inflammation reduction, oxidative stress prevention, and the inhibition of apoptosis, by precisely delivering therapeutic drugs and efficiently penetrating the BBB. Current evidence supports the efficacy of polymeric, lipid-based, and hybrid NPs in modulating the PI3K/AKT/CREB pathway; however, challenges remain regarding biocompatibility, long-term safety, and scalable production.

New strategies, such as biomimetic and smart NPs that respond to brain-specific factors such as oxidative stress and pH, offer promising avenues for improving therapeutic targeting and efficacy. Integrating NPs with gene therapy, stem cell treatments, or neurotrophic factors such as BDNF could enhance recovery. Furthermore, leveraging AI to refine NP design, analyze clinical data, and develop personalized treatment plans could transform stroke care entirely.

Despite these advances, significant hurdles persist. Comprehensive preclinical and clinical studies are needed to address long-term safety concerns, including potential toxicity, bioaccumulation, and off-target effects. Ensuring regulatory compliance for clinical use and scaling up production with consistent quality remain critical challenges. Furthermore, there is a lack of research on the use of NPs for the chronic phase of IS, in which neuroinflammation exerts significant influence on neurobehavioral impairment.

NPs also hold potential for treating neurological illnesses, including Alzheimer’s and Parkinson’s diseases. To advance these treatments from experimental to clinical stages, interdisciplinary collaboration is crucial. By facilitating targeted medication administration and activating the PI3K/AKT/CREB pathway, NPs have the potential to promote neuroprotection and recovery in IS. However, issues such as tissue accumulation, immunogenicity, and toxicity must be addressed. Thorough preclinical and clinical research is required to assess effectiveness, pharmacokinetics, and safety. Personalized nanomedicine, based on patient characteristics, could lead to standardized therapies for neurological illnesses and stroke. Because of their improved efficacy and tailored delivery, nanoparticle-based treatments have enormous potential to improve the treatment of stroke. Although non-biodegradable NPs may provide persistent toxicities and bioaccumulation issues, their clinical translation necessitates rigorous safety considerations. It is also necessary to address ethical issues such as equal access, environmental effect, and consent from patients. These challenges indicate the importance of comprehensive regulatory frameworks to ensure that nanoparticle-based therapies are both safe and equitable. By fostering collaboration between researchers, clinicians, and policymakers, we can work towards maximizing the benefits of these innovative treatments while minimizing potential risks. To guarantee thorough testing, oversight, and approval of nanomedicines, regulatory frameworks must change. The safe and successful application of NPs for stroke treatment requires a well-rounded strategy that combines creativity, moral obligation, and legal compliance.

## Data Availability

*Not applicable*.
